# The Role of Adjuvant Hormonal Treatment after Surgery for Localized High-Risk Prostate Cancer: Results of a Matched Multiinstitutional Analysis 

**DOI:** 10.1155/2012/612707

**Published:** 2012-01-31

**Authors:** Maria Schubert, Steven Joniau, Paolo Gontero, Susanne Kneitz, Claus-Jürgen Scholz, Burkhard Kneitz, Alberto Briganti, R. Jeffrey Karnes, Bertrand Tombal, Jochen Walz, Chao-Yu Hsu, Giansilvio Marchioro, Pia Bader, Chris Bangma, Detlef Frohneberg, Markus Graefen, Fritz Schröder, Paul van Cangh, Hein van Poppel, Martin Spahn

**Affiliations:** ^1^Department of Urology and Pediatric Urology, Comprehensive Cancer Center Mainfranken, University Hospital Würzburg, Oberdürrbacher Strasse 6, 97080 Würzburg, Germany; ^2^Department of Urology, University Hospitals Leuven, 3000 Leuven, Belgium; ^3^Department of Urology, University of Turin, 10126 Turin, Italy; ^4^Microarray Unit, University of Würzburg, 97078 Würzburg, Germany; ^5^Department of Urology, Vita-Salute San Raffaele University, 20132 Milan, Italy; ^6^College of Medicine, Mayo Clinic, Rochester, MN 55905, USA; ^7^Department of Urology, Université Catholique De Louvain, 1348 Brussels, Belgium; ^8^Department of Urology, University Medical Centre Hamburg-Eppendorf, 20246 Hamburg, Germany; ^9^Department of Urology, University of Piemonte Orientale, 28100 Novara, Italy; ^10^Department of Urology, Community Hospital Karlsruhe, 76133 Karlsruhe, Germany; ^11^Department of Urology, Erasmus MC, 3015 CE Rotterdam, The Netherlands

## Abstract

*Introduction*. To assess the role of adjuvant androgen deprivation therapy (ADT) in high-risk prostate cancer patients (PCa) after surgery. *Materials and Methods.* The analysis case matched 172 high-risk PCa patients with positive section margins or non-organ confined disease and negative lymph nodes to receive adjuvant ADT (group 1, *n* = 86) or no adjuvant ADT (group 2, *n* = 86). *Results*. Only 11.6% of the patients died, 2.3% PCa related. Estimated 5–10-year clinical progression-free survival was 96.9% (94.3%) for group 1 and 73.7% (67.0%) for group 2, respectively. Subgroup analysis identified men with T2/T3a tumors at low-risk and T3b margins positive disease at higher risk for progression. *Conclusion*. Patients with T2/T3a tumors are at low-risk for metastatic disease and cancer-related death and do not need adjuvant ADT. We identified men with T3b margin positive disease at highest risk for clinical progression. These patients benefit from immediate adjuvant ADT.

## 1. Introduction

Patients with high-risk localized prostate cancer (PCa) based on either PSA >20 ng/mL, Gleason score (GS) ≥8, or an advanced clinical stage have a risk of biochemical failure of up to 70% with surgery alone [1–5]. This has raised the question on the need of adjuvant treatments including androgen deprivation, radiation, and chemotherapy. Adjuvant androgen deprivation therapy (ADT) has shown significant improvement in disease-free survival for men with high-risk PCa treated with definitive radiation therapy and a survival benefit for men with GS 8–10 [6, 7]. For patients treated with radical prostatectomy (RP) the role of adjuvant ADT is still controversial. In a small prospective, randomized trial a survival benefit with adjuvant ADT in patients with lymph node positive disease was shown [8]. Two retrospective studies have reported a survival advantage for immediate ADT in patients with locally advanced disease [9, 10]. For patients with pT3N0M0 PCa Thompson et al. recently reported improved metastasis-free and overall survival (OS) with adjuvant radiation therapy when compared to observation [11]. Current guidelines therefore recommend adjuvant radiation for these patients [12, 13]. However, the results of the ADT-alone control arm of the SWOG study S9921 reported on excellent 5-year progression-free (92.5%) and OS rates (95.9%) for men with high-risk PCa treated with RP and adjuvant ADT over a two-year period [14]. These excellent results were seen despite a minority of patients receiving adjuvant radiation and therefore suggest there might be a role for adjuvant ADT in men with pT3 disease and/or positive surgical margin. 

The aim of our study was to analyze the role of adjuvant hormonal therapy in high-risk PCa patients with positive section margins or non-organ confined disease, but without nodal involvement, after radical prostatectomy in a matched European multicenter study cohort.

## 2. Materials and Methods

### 2.1. Patient Population

The study included 1413 patients with clinically localized high-risk PCa (PSA > 20 ng/mL, cT3-4, biopsy GS 8–10) and negative bone scan who had undergone RP at 7 tertiary referral centers between 1989 and 2005. Patients with positive section margins or non-organ confined disease and negative lymph nodes represented the study population. These patients were case matched in two groups receiving either adjuvant ADT within the first 3 months after RP (group 1) or no adjuvant ADT (group 2) (match criteria: age, clinical stage, biopsy and specimen Gleason score, pathological stage, and surgical margin status). Hormone deprivation was continuous and varied according to institutional preferences. Orchiectomy, LHRH-therapy, or maximal androgen deprivation with flutamide were performed. Neoadjuvant HT or adjuvant radiation therapy was considered as exclusion criteria. All patients were staged preoperatively with digital rectal examination (DRE), an abdominopelvic computed tomography (CT) scan, and bone scan. They underwent a wide radical prostatectomy with pelvic lymph node dissection. Our study group is not homogenous for the extent of lymphadenectomy and varied according to the institutional preferences, thus limiting our study in this point. Salvage therapy such as androgen deprivation (group 2 only) or radiotherapy was performed in individual patients at biochemical or clinical recurrence.

### 2.2. Postoperative Evaluation and Endpoints

Follow-up included a DRE and serum PSA analysis every 3 months for 2 years, every 6 months until 5 years after surgery, and annually thereafter. Imaging with US, CT scan, or bone scan was performed at the appearance of biochemical progression (BP), or manifest symptoms. BP was defined as PSA >0.2 ng/mL on 2 consecutive follow-up visits, clinical progression (CP) was either defined as local recurrence (confirmed by histology or imaging), or systemic recurrence (suspected by CT or bone scan). Prostate cancer-specific mortality (PCSM) was defined as the time from RP to death attributed to PCa or disease-related complications. Prostate cancer-specific survival (PCSS) was defined as the time from RP to PCSM. Overall survival (OS) was defined as the time from RP to death from any cause.

### 2.3. Statistical Analysis

 Biochemical progression-free survival (BPFS), clinical progression-free survival (CPFS), overall survival (OS), and cancer-specific survival (PCSS) from the time of surgery were defined as endpoints for this retrospective analysis. Continuous variables were summarized as the mean and standard deviation. The Kaplan-Meier method was used to estimate the survivor function at various time points. Group comparisons were made using the log-rank test for survival endpoints. Univariate hazard ratios and their 95% confidence intervals (CI) were estimated using the Cox proportional hazards model. Prespecified clinical variables considered in the Cox model included the preoperative serum PSA, biopsy Gleason score, and clinical stage. Pathological stage, specimen Gleason score, surgical margin status, lymph node involvement, and adjuvant ADT were used as postoperative parameters. A *P* value of <0.05 was considered statistically significant. Cox regression models were performed in order to identify subgroups of patients who either benefit from adjuvant ADT or never needed any ADT. Classification of subgroups was as follows: Gleason score <7/7/>7; positive and negative section margin pT2/T3/T4. Predefined endpoints were BP and CP. All analyses were performed with R statistical software (R, free software foundation). Multivariate analyses were insufficient to interpret since groups were too small.

## 3. Results

Out of 1413 patients 800 met the inclusion criteria. From these 86 were matched into each group. The homogeneity of both groups is shown in Table 1.

### 3.1. Biochemical Recurrence-Free Survival

At a median follow-up of 67 months 35.5% of the patients developed BP. BP was less frequent in group 1 when compared to group 2 (Table 2). The univariate Cox regression analyses are presented in Table 3.

### 3.2. Clinical Progression-Free Survival

Of the 86 patients in group 1 only 5 (5.8%) experienced CP during follow-up. On the contrary—although follow-up for clinical recurrence was available only for 46 patients in group 2—CP was seen in 12 of these 46 patients available for analysis (26.1%). Estimated 5- and 10-year CPFS 96.9% and 94.3% for group 1 and 73.7% and 67.0% for group 2, respectively (*P* < 0.01) (Table 2). None of the men with T2 tumors developed CP, and the risk for T3a disease was fairly low (2/28 in group 1 versus 4/29 in group 2, *P* = 1.0). But all patients who developed CP had positive section margins. The risk was highest in men with seminal vesical invasion (T3b) and univariate cox regression analysis comparing both groups showed that tumor stage, surgical margin status, and Gleason score were predictors for CP (Table 3). The numbers were too small for multivariate cox regression analysis.

### 3.3. Cancer-Specific Survival and Overall Survival 

Survival for the entire cohort was excellent. There have been 20 deaths (11.6%) including only 4 PCa-related deaths (2.3%). Although there was no statistically significant difference for OS and PCSS between both groups none of the patients in group 1 died PCa-related while 4 in group 2 died on their prostate cancer. The estimated 10-year. OS and PCSS was 83.8% and 100% for group 1 and 76.4% and 91.0% for group 2, respectively (Table 2).

## 4. Discussion

There is increasing evidence that surgery provides a reasonable treatment option for selected men with high-risk prostate cancer [1, 5, 15, 16]. The recently reported results of the control arm of the SWOG-study S9921 showed that the combination of surgery and combined adjuvant ADT is associated with favorable disease-free and overall survival of greater than 92% at 5 years of follow-up [14]. Our study results corroborate these better than expected survival rates even for a high-risk cohort with positive section margins or non-organ confined disease and negative lymph nodes (8-year PCSS 97.5% and OS 92.7%). The results reported here and in the S9921-trial together with the improved outcomes for the combination of radiation and ADT in men with high-risk prostate cancer support the use of a multimodal treatment including adjuvant ADT [7, 8, 17]. However, the survival rates reported in these trials reach up to 90% for PCSS and 76% for OS, indicating that what we currently define as “high-risk” disease group indeed is a heterogeneous cohort with better than expected outcomes. These limitations in risk assessment are also visible in the adjuvant radiation therapy (RT) trials. Although some differences exist among the inclusion criteria, these studies showed a benefit for immediate adjuvant radiation in terms of biochemical progression (hazard ratio 0.47, 95% CI: 0.4–0.56, *P* < 0.0001) [17]. But only the SWOG-study could show a significant improvement in metastasis-free and OS of 1.8 and 1.9 years, respectively [11]. Overtreatment is obvious from these RT trials: the number needed to treat was 12.2 to prevent metastasis in one patient at 12.6 years of follow-up and the number needed to treat was 9.1 to prevent one death at the same time. Therefore, Colette et al. tried to substratify the patients from EORTC-trial 22911 and identified men with positive section margins to be at higher risk for biochemical progression (relative risk reduction of 62% for irradiated men, HR 0.38) [18].

The excellent outcomes observed in men receiving adjuvant ADT raise two questions. (1) Overtreatment; the results of the control arm of the S9921-trial show better than expected survival rates with adjuvant ADT over a two-year period [14]. These excellent results raise the question whether adjuvant ADT was necessary in all patients after surgery. ADT is related to a wide variety of metabolic and cardiovascular effects that impact morbidity and mortality of PCa patients [19]. Treatment deescalation therefore is an important step forward in treating men with PCa. Our matched analysis showed that 43% of the patients treated by RP alone never experienced BP during follow-up and the estimated 10-year biochemical progression-free survival for this group was 30.6%. However, PSA is limited as an outcome parameter and not all patients with biochemical recurrence will develop metastasis and finally die on their PCa thus limiting this information in patients counseling [20, 21]. Analysis therefore should be focused on clinical progression and survival. Although our data for group 2 are limited in terms of clinical recurrence—follow-up information for this outcome measure were available only for 46/86 men in this group—the risk for CP was significantly lower for group 1 when compared to group 2 (5.7% versus 26.1%). Men with T2 and T3a tumors (irrespective to the surgical margin status) had a very low risk of CP and PCSM and therefore adjuvant ADT can be avoided in these patients. (2) The excellent outcomes make compelling argument for a better definition of high-risk patients for future trials. We identified tumor stage, surgical margin status, and Gleason score as predictors for CP. Men with T3b margin positive disease are at increased risk for CP and benefit from adjuvant ADT with a hazard ratio of 0.25 when comparing group 1 and 2. EAU guidelines state two options that can be offered to patients with pT3b tumors and positive margins: either an immediate radiotherapy to the surgical bed, upon recovery of urinary function, or monitoring followed by salvage radiotherapy at PSA rising, not exceeding 0.5 ng/mL (level of evidence 1 and 3) [12]. However, this trial was conducted to assess the role of adjuvant ADT in high-risk PCa patients after surgery. Since this study lacks a radiotherapy arm, comparison to other adjuvant therapy regimens cannot be drawn. Side effects, costs, and benefits have to be considered when deciding on adjuvant therapy. In our study survival rates were excellent—only 11.6% of the patients died overall and 2.3% tumor related. Although not statistically significant some differences in the outcome between both treatment groups have to be mentioned. None of the patients in group 1 died tumor related versus 4 in group 2. All of these 4 men had positive surgical margins and 3/4 T3b tumors, thus suggesting that these patients might benefit from immediate adjuvant ADT. In general, the risk of death is rather low even for the group of T3b patients. Further subanalyses are necessary to identify those men at highest risk for CP and PCSM. It is important to mention that all patients in the adjuvant ADT group received continuous ADT. It is therefore not possible to address the question whether this approach is superior to shorter-term adjuvant ADT or PSA-triggered ADT. In a retrospective study Siddiqui et al. reported on the outcome of a contemporary RP cohort of 6401 men and compared the outcome of immediate-versus deferred PSA-triggered ADT and found no differences in CSS [22].

We recognize that our study is not without limitations. Its retrospective nature may have caused a selection bias towards patients who, although considered high risk, were still deemed suitable for surgery. Variations in the extent of lymphadenectomy and in pathology review as well as the use of salvage treatments might have influenced our results. The study design and the low number of events (clinical recurrence, and PCSM) limit the power of the analysis.

Despite these limitations, the results of this matched multicenter study on RP and adjuvant ADT provide useful information for clinical decision making for men with high-risk prostate cancer and adverse histopathological parameters.

## 5. Conclusion

Our results indicate excellent outcomes for high-risk prostate cancer with positive section margins or non-organ confined disease but negative lymph nodes after surgery. Patients with T2/T3a tumors are at low risk for metastatic disease and cancer-related death even in case of positive section margins—adjuvant ADT therefore can be avoided in these patients. Pathological stage and section margin status allows us to identify men with T3b surgical margin positive disease at highest risk for clinical progression. These patients benefit from immediate adjuvant ADT. However, such risk stratification is limited and far away from personalized therapy. Research energy should be focused on the identification and validation of new molecular markers to identify lethal disease. We recently described a new biomarker to predict clinical recurrence in high-risk PCa patients [23].

## Figures and Tables

**Table 1:** Preoperative and postoperative characteristics of the 172 matched patients.

Characteristic	Group 1	Group 2
	Adjuvant ADT	No adjuvant ADT
Patients number	86	86
Median age (years)	66.2	66.6
Median Follow-up (months)	69	66
Mean PSA, ng/mL (range)	31.5 (3–119)	28.4 (2.78–159)

Clinical stage, 1997 TNM, (number (%))		
≤cT2	36 (41.8%)	48 (55.8%)
cT3	49 (57.0% )	37 (43.0%)
cT4	1 (1.2 %)	1 (1.2%)

Biopsy Gleason score (number (%))		
≤6	36 (41.8%)	44 (51.2%)
7	39 (45.3%)	32 (37.2%)
≥8	11 (12.9%)	10 (11.6%)

Pathol. stage, 1997 TNM (number (%))		
pT2	4 (4.7%)	9 (10.5%)
pT3a	28 (32.5%)	29 (33.7%)
pT3b	50 (58.1%)	45 (52.3%)
pT4	4 (4.7%)	3 (3.5%)

Pathol. Gleason score (number (%))		
≤6	34 (39.5%)	34 (39.5%)
7	39 (45.4%)	39 (45.4%)
≥8	13 (15.1%)	13 (15.1%)

Surgical margins (number (%))		
Positive	62 (72.1%)	66 (76.7%)
Negative	24 (27.9%)	20 (23.3%)

Salvage therapy		
ADT	0 (0.0%)	25 (29.1%)
Radiotherapy	3 (3.5%)	9 (10.5%)

PSA: prostate-specific antigen; ADT: androgen deprivation therapy.

**Table 2:** Freedom from biochemical progression free-survival (BPFS), clinical progression-free (CPFS), cancer-specific (CSS), and overall survival (OS).

Projected survival	Group 1 ADT (*n* = 86)		Group 2 no ADT (*n* = 86)		*P* value
	5 years	10 years	5 years	10 years	
BPFS	87.7%	76.3%	37.1%	30.6%	<0.001
CPFS	96.9%	94.3%	73.7%	67.0%	0.003
CSS	100%	100%	100%	91.0%	0.9
OS	94.4%	83.8%	97.1%	76.4%	0.6

**Table 3:** Univariable cox regression models for comparing groups 1 and 2; endpoint: biochemical recurrence and clinical progression. (For clinical progression follow-up data were available for 132 patients).

	Biochemical progression		Clinical progression	
	HR (95% CI)	*P* value	HR (95% CI)	*P* value
Whole sample	0.15 (0.08–0.29)	<0.001	0.21 (0.08–0.6)	0.003
Low Gleason <7	0.16 (0.08–0.31)	<0.001	0.25 (0.08–0.8)	0.02
High Gleason ≥7	0.11 (0.01–0.86)	0.04	0.18 (0.02–1.6)	n.s.
R0	0.13 (0.03–0.48)	0.002	1.15 (0.1–13.4)	n.s.
R1	0.16 (0.75–0.32)	<0.001	0.15 (0.04–0.53)	<0.01
pT3a	n.s.	n.s.	n.s.	n.s.
pT3b	0.15 (0.08–0.29)	<0.001	0.26 (0.09–0.75)	0.013
pT4	0.22 (0.02–2.1)	n.s.	n.s.	n.s.
